# The practice of internet hospital for pediatric patients: a real-world study

**DOI:** 10.3389/fped.2025.1562127

**Published:** 2025-10-23

**Authors:** Wen Yin, Zhuo Li, Chenzhong Gao, Jin Xu, Qian Wang, Wenjing Li

**Affiliations:** ^1^ Department of Outpatient Office, Children’s Hospital of Nanjing Medical University, Nanjing, China; ^2^ Department of Emergency/Critical Medicine, Children’s Hospital of Nanjing Medical University, Nanjing, China; ^3^ Department of Pharmacy, Children’s Hospital of Nanjing Medical University, Nanjing, China; ^4^ Department of Administrator’s Office, Children’s Hospital of Nanjing Medical University, Nanjing, China

**Keywords:** internet hospital, pediatric patients, online follow-up services, online consultation services, pediatric chronic disease management

## Abstract

**Objective:**

The study analyzes real-world operational data of our pediatric internet hospital to improve operational efficiency, enhance user experience, and provide a reference for other hospitals launching similar platforms.

**Methods:**

We analyzed the operational data of our pediatric internet hospital from June 1, 2020, to May 31, 2025, examining patient demographics, service utilization, diagnoses, and satisfaction metrics. We also assessed the platform's key modules-medical assistant services, online treatment, and chronic disease management.

**Results:**

Over five years, the internet hospital registered 2,096,293 new users. A total of 259,034 patients used online follow-up services, with 175,654 prescriptions issued, while 266,321 patients utilized online consultation services. The majority (76.27%) of follow-up patients were under six years old, and services expanded to 31 provinces by 2025. Patient satisfaction was high, with an average score exceeding 9 out of 10. However, limitations in online physical examinations and diagnostic accuracy, along with policy-related restrictions, pose challenges to further expansion.

**Conclusions:**

Internet hospitals offer a valuable supplement to offline care, enhancing accessibility, particularly for children in remote areas. Despite certain operational limitations, internet hospitals have the potential to transform healthcare delivery in China. Further efforts should focus on expanding specialty services and addressing policy barriers to maximize their impact.

## 1 Introduction

The internet hospital is an innovative healthcare model that has evolved from the “Internet + Health Care” framework, extending the principles of telemedicine and traditional hospital care (1). While telehealth or telemedicine models have been studied and implemented for many years in regions like the European Union, the United States, and Japan, they have typically been applied in specialized fields such as ophthalmology, cardiovascular care, and dermatology (2). In contrast, China, with its large population and uneven distribution of healthcare resources, requires a more advanced model that extends the scope and accessibility of healthcare beyond conventional telehealth (3). As of August 2021, over 1,600 internet hospitals in China have received official government licenses, however, these platforms primarily cater to adult patients, especially those with common or chronic diseases, as well as those in rural or underserved areas.

As one of the leading tertiary pediatric hospitals in China, our institution launched the first large-scale internet hospital for children in Jiangsu Province on June 1, 2020, with full operations commencing in July 2020. Over the past three years, we have accumulated extensive experience in the operation and management of internet hospitals specifically designed for children. This study aimed to provide a comprehensive overview of our platform's construction, operational model, functional positioning, treatment processes, day-to-day management, and real-world patient experiences with internet hospitals.

## 2 Methodology

### 2.1 General information

This study retrospectively analyzed the data of internet hospital of Children's Hospital of Nanjing Medical University from June 1, 2020, to May 31, 2025. This retrospective study was approved by the Medical Ethics Committee of Children's Hospital of Nanjing Medical University, and informed consent was waived due to the de-identified nature of the data.

### 2.2 Inclusion and exclusion criteria

#### 2.2.1 Inclusion criteria

Users who registered on the internet hospital including users utilized the medical assistant services and users engaged in online treatment and chronic disease management services.

#### 2.2.2 Exclusion criteria

(1) Users who registered on the internet hospital, but did not utilize any services;
(2) Patients with incomplete information.

### 2.3 Platform construction and operations management

**Figure 1:**
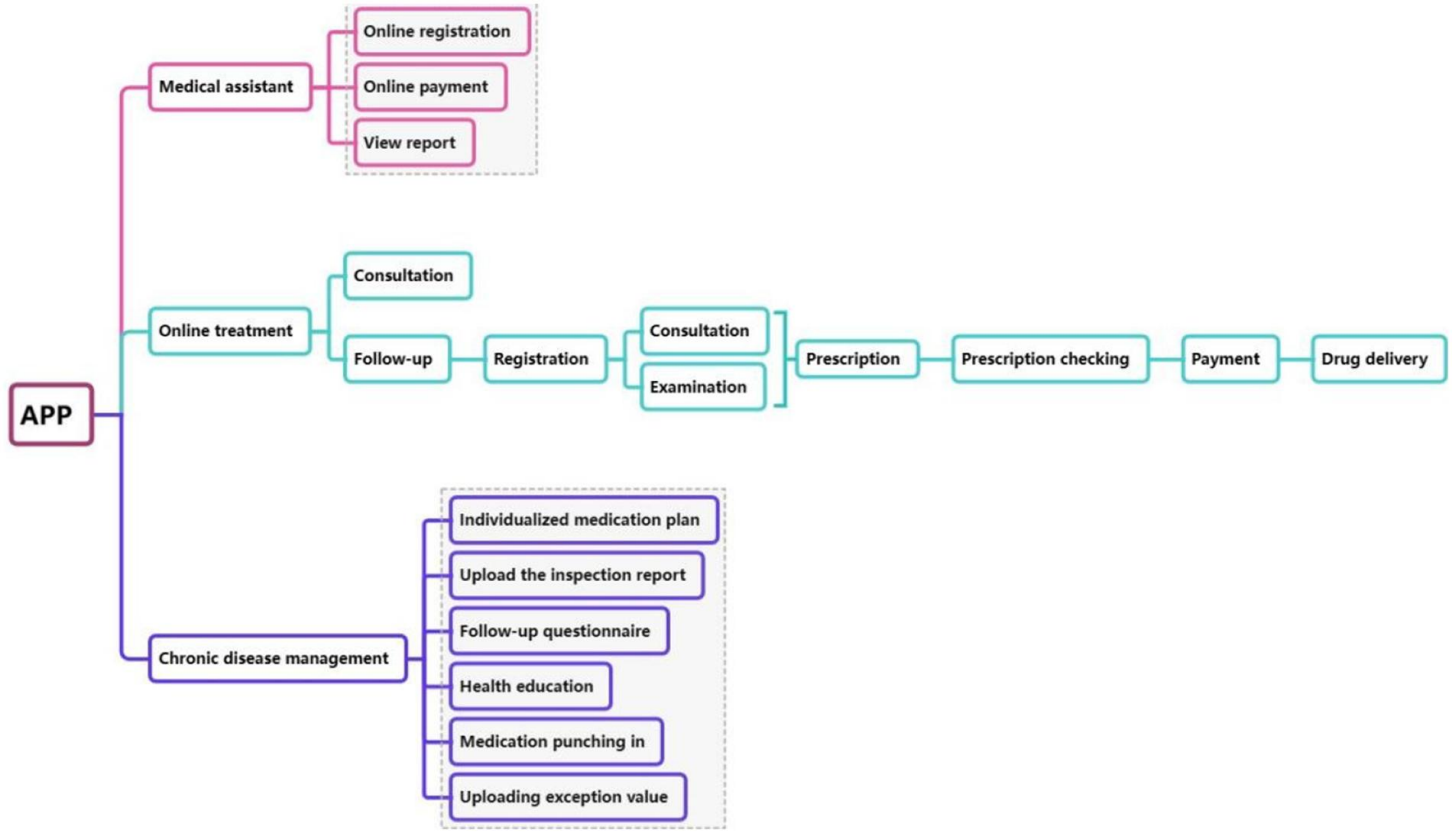
Platform construction of the system.

The construction of the internet hospital platform follows a hierarchical integration approach, and is organized into three main modules using the microservice architecture: medical assistant module, online treatment module and chronic disease management module. Regarding platform architecture, the internet hospital was developed based on a microservice architecture to optimize system stability and security. Each functional module was designed as an independent service, allowing for flexible scaling, load balancing, and fault isolation. This structure reduces the risk of system crashes during high user traffic. Furthermore, data synchronization mechanisms ensure seamless integration with the hospital information system (HIS). To safeguard pediatric patient privacy, multiple layers of data encryption, role-based access control, and real-time monitoring were implemented in compliance with national data security and healthcare privacy regulations. The overall system architecture is shown in Figure 1.

The medical assistant module incorporates traditional functions from the hospital's existing system, such as appointment registration, online payment, and viewing inspection reports. The online treatment module serves as a crucial supplement to offline medical services and is considered the core module of the internet hospital for children. It includes two key services: online consultations and online follow-up visits. Online consultations are conducted by experts who provide professional advice and guidance to patients. However, prescriptions cannot be issued during these consultations, and there are no fixed schedules for patient-doctor interactions. Patients can ask questions freely and even leave follow-up messages after the consultation has concluded. In contrast, online follow-up visits are scheduled in advance and allow for prescription of medications and medical procedures. All online prescriptions undergo a stringent review process by pharmacists. After verification in the HIS system and ensuring appropriate drug use, the prescription is approved, allowing the patient to proceed with payment and arrange drug delivery. For children with chronic diseases, the internet hospital integrates dedicated chronic disease management modules. These modules include health education, medication tracking, the development of individualized medication plans, follow-up questionnaires, and the ability to upload inspection reports and abnormal results. Even after discharge, children with chronic conditions can receive timely guidance and assistance from medical experts, ensuring continuous care throughout the treatment cycle.

### 2.4 Collection of data

Data were collected, including age, gender, address, diagnosis, medical expenses, visit time and patient satisfaction.

### 2.5 Statistical methods

Statistical analyses were performed using SPSS 26.0 software. Continuous variables following a normal distribution were expressed as mean ± standard deviation (x¯ ± s), while categorical variables were presented as frequency and percentage [*n* (%)]. The *t*-test was used for comparisons between groups, and categorical data were analyzed using the *χ*² test. A *P*-value < 0.05 was considered statistically significant. A two-sided *P* < 0.05 was considered statistically significant.

## 3 Results

### 3.1 Overall utilization of the internet hospital

Since the launch of our internet hospital five years ago, more than 2,096,293 new users registered on the platform, among which, 259,034 patients received online follow-up services, resulting in the issuance of 175,654 online prescriptions, 266,321 patients accessed the online consultation services, while the number of offline visits during the same period reached 14,458,261.

From June 1, 2020 to May 31, 2025, the number of patients using the internet hospital increased by approximately 50% year-on-year. In order to better understand the participation of patients with different types of services, we conducted statistical analysis on the demographic data, distribution of patient regions, distribution of diagnoses, medical expenses, visit time and patient satisfaction, compared them with offline visit data during the same period, and wrote monthly reports to track online and offline visit situations. These comprehensive data comparisons play a vital role in continuously improving the services of Internet hospitals, and help optimize patient care and operational efficiency.

### 3.2 Demographic data

Among the 259,034 follow-up patients, males accounted for 54.71%, while among the 266,321 consultation patients, males represented for 55.59%. Similarly, among the offline patients during the same period, males constituted 57.07%. These findings suggest that the gender distribution of all patient groups showed a slight male predominance, which was more pronounced in patients presenting offline, but the differences were minimal.

Patients were categorized into two age groups: those younger than 6 years and those 6 years and older. Among the follow-up patients, children under 6 years old accounted for 76.27%, and among consultation patients, the proportion of children under 6 years old accounted for 76.66%. In comparison, only 50.40% of patients who visited offline during the same period were under the age of 6 years old. This indicates that patients under 6 years old tend to choose online medical treatment mode. The higher proportion of patients under the age of 6 years old across all groups highlights the critical role internet hospitals play in managing children, particularly young children who may face more frequent health concerns and require constant monitoring. The results are shown in Table 1.

**Table 1:** Demographic data of patients in internet hospital and offline patients in the same period.

Characteristic	Follow-up patients	Consultation patients	Offline patients
Total	259,034 (100.0)	266,321 (100.0)	14,458,261 (100.0)
Gender			
Male	141,712 (54.71)	148,047 (55.59)	8,251,731 (57.07)
Female	117,322 (45.29)	118,274 (44.41)	6,206,530 (42.93)
Age			
≤6 years	197,563 (76.27)	204,163 (76.66)	7,287,208 (50.40)
>6 years	61,471 (23.73)	62,158 (23.34)	7,171,053 (49.60)

### 3.3 Distribution of patient regions

**Figure 2:**
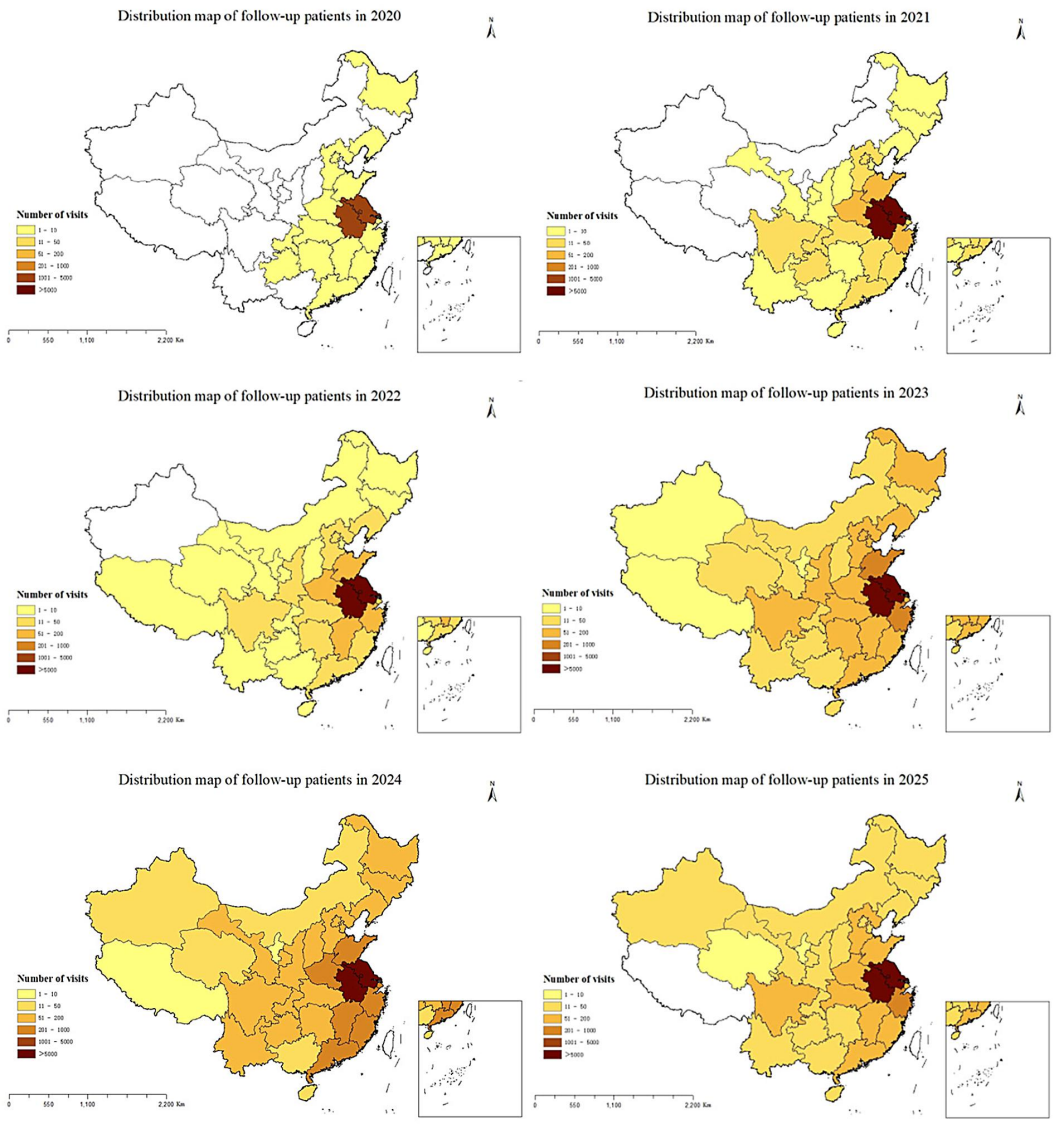
The regional distribution of follow-up patients from 2020 to 2025.

**Figure 3:**
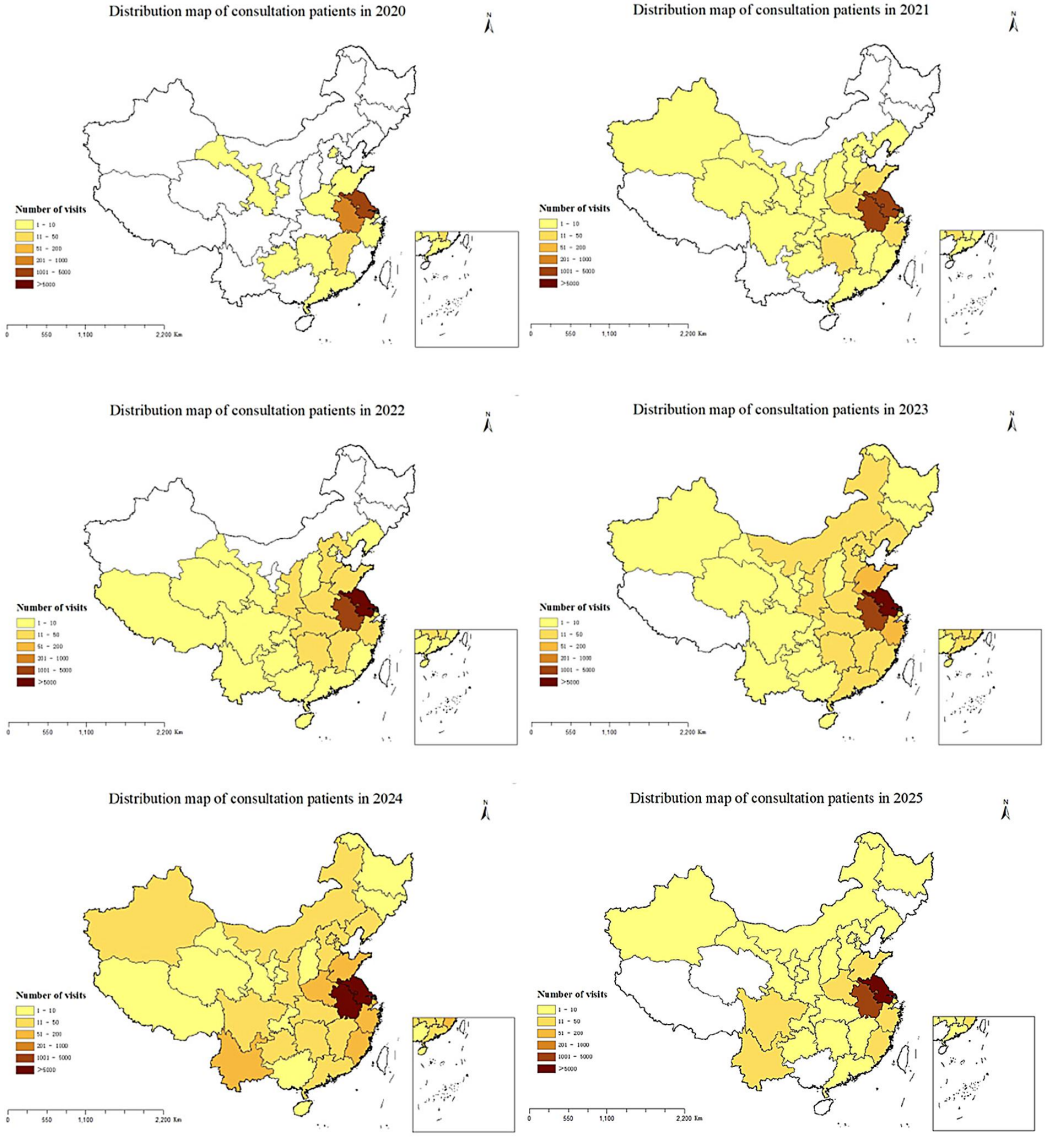
The regional distribution of consultation patients from 2020 to 2025.

All follow-up patients provided detailed addresses, and the number of provinces covered by follow-up services increased from 17 in 2020 to 31 in 2025, with the number of follow-up patients showing an upward trend year by year. The regional distribution of follow-up patients from 2020 to 2025 is shown in Figure 2. Only 70,876 (26.61%) of the 266,321 consultation patients provided the addresses, although the proportion of those who registered the address is relatively low, the geographical distribution has increased from 12 provinces in 2020 to 31 provinces, autonomous regions, and municipalities. Similar to the follow-up patients, the number of consulting patients has been increasing year by year. The regional distribution of consultation patients from 2020 to 2025 is shown in Figure 3.

**Figure 4:**
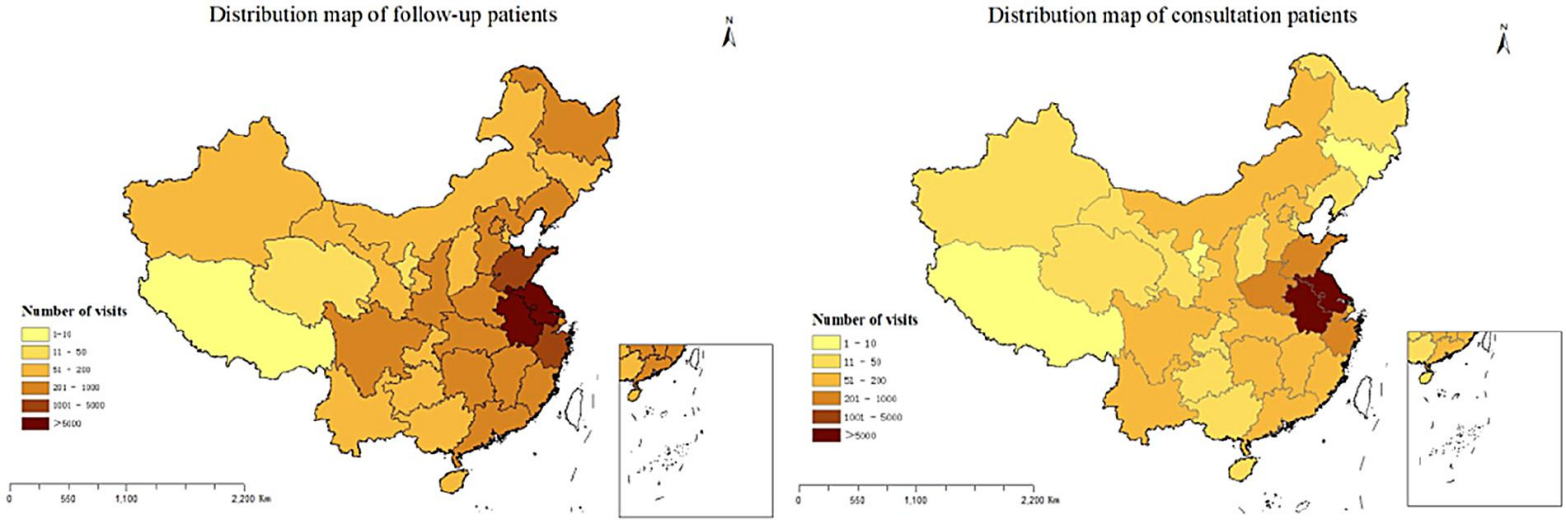
The overall regional distribution of follow-up and consultation patients from 2020 to 2025.

**Figure 5:**
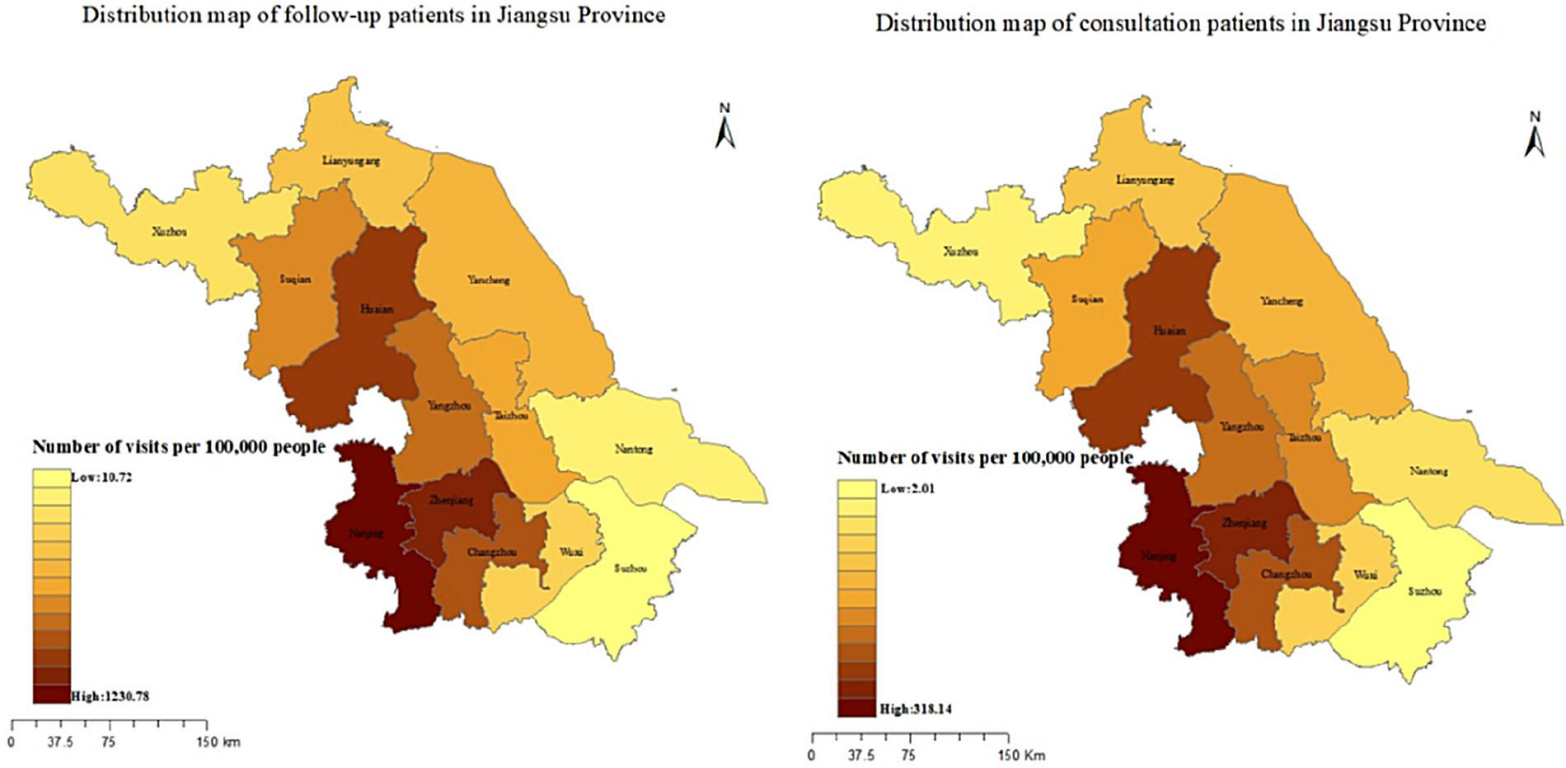
The overall geographical distribution of follow-up and consultation patients from 2020 to 2025 in Jiangsu province.

**Figure 6:**
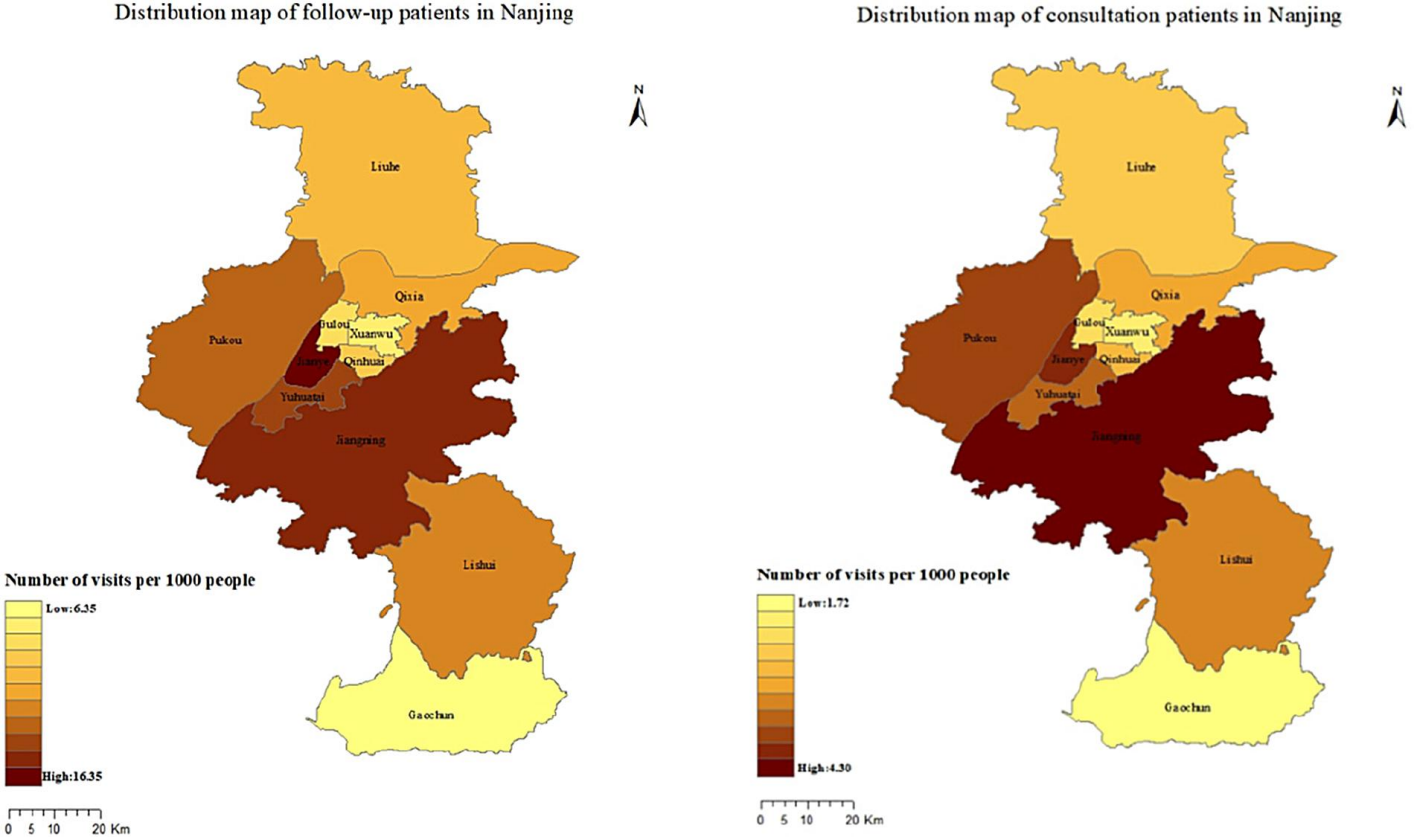
The overall geographical distribution of follow-up and consultation patients from 2020 to 2025 in Nanjing.

The majority of the follow-up and consultation patients were from Jiangsu Province, followed by Anhui Province. The overall geographical distribution of patients is shown in Figure 4. We found that 57.64% of consultation patients and 61.35% of follow-up patients were from Nanjing, followed by Zhenjiang, Changzhou and Huai'an, the fewest patients came from Suzhou. Figure 5 shows the distribution of the number of patients by city. Further analysis of the distribution of patients in different districts of Nanjing showed that Jiangning District had the largest number of patients, followed by Pukou District, Luhe District and Qixia District district, both for consultation and follow-up, Gaochun District has the lowest number of cases, as shown in Figure 6.

### 3.4 Distribution of diagnoses

As consultation patients did not receive a specific diagnosis, our analysis focused on the distribution of diagnoses among follow-up patients. Respiratory system diagnosis accounted for the majority, reaching 47.59%, followed by follow-up visits primarily for prescription refills, with no new diagnostic assessment. The drugs prescribed for this group of patients were commonly used respiratory system drugs for children and drugs that required long-term use such as antiepileptics. The remaining 23.33% of patients received other diagnoses.

Our internet hospital currently offered chronic disease management services for children with conditions such as acute lymphoblastic leukemia, asthma, and epilepsy. These services were delivered by specialists who are trained to manage these specific diseases, either through online consultations or follow-up visits.

### 3.5 Medical expenses

Among the follow-up patients, for those who did not receive a prescription, medical expenses only include registration fees, while for those who received a prescription, medical expenses include both registration fees and medication costs. The registration fee varied according to the professional title of the doctor: 12 yuan, 22 yuan, and 35 yuan for resident physicians, associate chief physicians, and chief physicians, respectively. Most follow-up patients (88.20%) chose resident doctors, whereas fewer (11.80%) opted for higher-level doctors. This suggests that patients seeking only follow-up care may be more inclined to choose offline treatment when higher-level expertise is needed. Prescription analysis revealed that the majority of the drugs in the prescription were commonly used for pediatric respiratory infections, with a cost typically below 200 yuan. The remaining drugs in the prescription were for treating chronic diseases such as epilepsy, convulsive disorders, and asthma, the cost of these drugs was usually over 200 yuan. Internet hospital consultation service only provided consultation without prescribing drugs. The consultation fees were set at 22 yuan for associate chief physicians and 35 yuan for chief physicians according to the doctor's professional title. 77.12% of consultation patients chose the option of 35 yuan, which was significantly higher than those patients who chose the option of 22 yuan, and there is a significant difference between the two cost options (*P* < 0.001).

### 3.6 Visit time

While many internet hospitals operated during the same hours as offline hospitals, our internet hospital provided services 24 h a day, allowing patients to seek medical assistance at any time. Notably, 51.44% of patients accessed services during non-working hours, and 61.91% of doctors provided consultations during these hours, ensuring that patients have consistent access to healthcare outside of traditional hours.

### 3.7 Patient satisfaction

After completing the consultation or follow-up service, the system will automatically prompt the patient to fill out a satisfaction questionnaire and rate the doctor's performance. The rating range is from 1 to 10, with 1 representing extreme dissatisfaction and 10 representing high satisfaction. To date, the satisfaction rates for our consulting and follow-up services have been 99.79% and 99.38%, respectively, with the average satisfaction score for doctors consistently above 9 points.

## 4 Discussion

In China, internet hospital serves as online medical platforms where medical institutions provide direct services for patients. By using the medical resources of the physical hospital and combining the internet technology, the coverage of the physical hospital is extended to the virtual space, and comprehensive services are provided in the closed-loop system, including medical consultation, prescription fulfillment and patient support, so that patients can obtain medical services in a comfortable home (4). Internet hospitals optimize the allocation of medical resources, improve the accessibility of medical services, and effectively combine the convenience of digital platforms with the comprehensive services of traditional hospitals (5). It is worth noting that during the COVID-19 pandemic (6), internet hospitals played a key role in infection control and could continue to provide medical services under strict social distance measures. They support self-care practices, promote epidemiological screening, and provide basic medical services to the public (7, 8). Most internet hospitals were established by medical institutions (9). Public tertiary hospitals, in particular, were the primary drivers behind the establishment of internet hospitals (10). Internet hospitals have rapidly developed in China as a healthcare innovation, but their use in pediatric care remains understudied.

Our internet hospital integrated three essential medical services-medical assistant, online treatment, and chronic disease management into a unified platform. This unified entry point, along with the centralized management of user, health, and order centers, significantly enhances patient convenience. By integrating these three modules (11), the internet hospital provided a wide range of services for children. It combined the convenience of online consultations with the professional support of expert follow-up care. The chronic disease management module enhanced the continuity of care, offering tailored support to children throughout the entire treatment process.

However, much of the existing literature on internet hospitals have focused on infrastructure development and the design of service models (12), and there are few research data on the real world.

Over the past five years, analysis of real-world patient data revealed that our internet hospital, as China's first specialized pediatric platform, had extended its services to nearly all provinces nationwide. This broad reach had facilitated the remote diagnosis and treatment of common pediatric conditions, particularly for patients in surrounding provinces like Jiangsu. The platform has been especially beneficial for children under six years old, addressing a critical need in pediatric healthcare. The availability of essential pediatric medications, coupled with an efficient drug delivery service, had minimized the need for long-distance travel, particularly during the COVID-19 pandemic.

Most follow-up patients chose to be followed up by a resident doctor because their main purpose of visit was to prescribe medication and consult simple questions. And consulting services focused more on the consultation of medical conditions and treatment plans, therefore, a relatively high proportion of patients chose high-level doctors (13).

Preliminary data from the chronic disease management module demonstrated encouraging trends in supporting medication adherence and chronic disease management for pediatric asthma and epilepsy. For example, real-time health education and medication tracking have been shown to improve treatment adherence in children with asthma, reducing the frequency of emergency visits. The ability to upload inspection reports and receive immediate feedback from specialists ensures that patients received timely and continuous care, contributing to better overall health outcomes. However, due to the lack of systematically collected quantitative adherence metrics, these findings should be interpreted as preliminary.

Previous studies have reported higher patient satisfaction with internet hospital services compared to traditional offline care (14), and our experience aligns with these findings (online 99.59% vs. offline 98.14%). The 24-hour availability of services enabled flexible scheduling for both patients and doctors, promoting effective communication and streamlined medical processes. These factors have contributed to improved patient experiences and a high level of satisfaction. This satisfaction scoring system allowed us to better understand patient needs and continually improve the quality of our services. Moreover, patients can use these scores as a reference when selecting doctors for future visits. This acceptance of internet hospitals as a legitimate healthcare model highlights their value as a complement to traditional healthcare systems.

The findings of this study are significant not only for pediatric care but also for the broader healthcare system, as they demonstrate how internet hospitals can be effectively integrated with physical hospital systems. The successful implementation of this model for children provides a blueprint that can be adapted for rural and underserved areas. Additionally, the adoption of this model could lead to advancements in telemedicine, such as the incorporation of AI-driven diagnostics and the use of wearable health technologies, further enhancing patient outcomes and healthcare accessibility.

## 5 Limitations

Despite the promising findings, our internet hospital model has several limitations. First, essential medical procedures, such as physical examinations, laboratory tests, and imaging, cannot be performed online, which may compromise diagnostic accuracy. Second, some patients continue to prefer traditional offline healthcare, and certain specialties are inherently unsuitable for full online delivery, thereby restricting the scalability of internet hospitals (15). Third, although we conducted logistic regression analysis, these were limited by the absence of key socioeconomic variables, such as regional healthcare resource disparities and caregiver education level, which may influence healthcare-seeking behaviors. Future studies with larger samples and more comprehensive data are needed to clarify the determinants of online vs. offline service preferences. Fourth, in the field of chronic disease management such as asthma and epilepsy, our study demonstrated encouraging trends toward improved adherence, but we were unable to provide quantitative outcome indicators such as adherence rates, emergency visits, or hospital readmissions. Incorporating standardized measures such as prescription refill adherence, validated adherence scales, and clinical endpoints will be in future evaluations. Fifth, the current platform is primarily hospital-based and has not yet integrated resources from primary healthcare institutions, limiting its broader regional reach. Medication distribution also faces logistical challenges: for example, cold chain and psychotropic drugs cannot yet be dispensed online. To address these issues, we plan to partner with licensed third-party logistics providers and explore innovative service models for prescription fulfillment. Finally, policy and infrastructure barriers remain. Limited medical insurance coverage for online follow-ups hinders widespread adoption, while primary care institutions in many regions lack the infrastructure to integrate internet hospital services. Moving forward, partnerships with medical associations, technology firms, and policymakers will be crucial to expand service coverage, promote resource sharing, and enhance capacity building. In addition, exploring advanced tools such as remote diagnostics and AI-assisted consultations may further overcome current limitations and enhance the scope of pediatric internet hospitals.

## 6 Conclusions

To the best of our knowledge, this study is the first to comprehensively explore the analysis of operational data from Internet hospitals dedicated to children, and the results show that internet hospitals are an effective and valuable complement to traditional offline healthcare, offering significant convenience to patients and optimizing the use of social resources. Given these benefits, the promotion and wider implementation of internet hospitals should be encouraged across healthcare institutions at various levels.

## Data Availability

The original contributions presented in the study are included in the article/Supplementary Material, further inquiries can be directed to the corresponding authors.
